# Reproductive factors and cognitive impairment in natural menopausal women: A cross-sectional study

**DOI:** 10.3389/fendo.2022.893901

**Published:** 2022-08-01

**Authors:** Haitao Xi, Jinghuan Gan, Shuai Liu, Fei Wang, Zhichao Chen, Xiao-Dan Wang, Zhihong Shi, Yong Ji

**Affiliations:** ^1^ Department of Neurology, Beijing Tiantan Hospital, Capital Medical University, Beijing, China; ^2^ Department of Neurology, Beijing Rehabilitation Hospital, Capital Medical University, Beijing, China; ^3^ Department of Neurology, Tianjin Dementia Institute, Tianjin Key Laboratory of Cerebrovascular and Neurodegenerative Diseases, Tianjin Huanhu Hospital, Tianjin, China; ^4^ Department of Neurology, Yuncheng Central Hospital of Shanxi Province, Yuncheng, China

**Keywords:** reproductive period, parity, mild cognitive impairment, dementia, Alzheimer’s disease

## Abstract

**Introduction:**

Little information on rural older women in northern China has been reported, apart from three studies in southern and eastern China in the past decade. This study aims to evaluate the relationships between reproductive factors and the risk of cognitive impairment, including mild cognitive impairment (MCI) and dementia, in Chinese women with natural menopause.

**Methods:**

The cross-sectional study was conducted in 112 community primary healthcare centers in rural northern China between April 2019 and January 2020. A total of 4,275 women aged ≥65 years who had natural menopause were included. Reproductive factors as well as the reproductive period (= age at menopause − age at menarche) were recorded. The relationships between reproductive factors and cognitive impairment were evaluated by correlation and logistic regression analysis.

**Results:**

Overall, 28.6% and 11.4% of women were diagnosed with MCI or dementia, respectively. In natural menopause women, the age at menopause (adjusted *r* = 0.070, *p* < 0.001), reproductive period (adjusted *r* = 0.053, *p* = 0.001), and number of pregnancies (adjusted *r* = −0.042, *p* = 0.007) and parities (adjusted *r* = −0.068, *p* < 0.001) were correlated with Mini-Mental State Examination (Chinese version) scores, and with similar findings concerning MCI and dementia with Lewy bodies (DLB). Greater age at menopause and a long reproductive period significantly decreased the risk of MCI and Alzheimer’s disease (AD), and more parities significantly increased the risks of MCI (odds ratio (OR) = 1.111, 95% confidence interval (CI): 1.039–1.187, *p* = 0.002), dementia (OR = 1.162, 95% CI: 1.061–1.271, *p* = 0.001), particular AD (OR = 1.131, 95% CI: 1.010–1.266, *p* = 0.032), DLB (OR = 1.238, 95% CI: 1.003–1.528, *p* = 0.047), and vascular dementia (VaD) (OR = 1.288, 95% CI: 1.080–1.536, *p* = 0.005).

**Conclusions:**

The prevalence rates of MCI and dementia were 28.6% and 11.4% in older women. Greater age at menarche, young age at menopause, shorter reproductive period, and larger numbers of pregnancies/parities were correlated with poor cognition and significantly increased the risks of MCI and dementia, particularly AD, DLB, and VaD.

## Introduction

With the aging of the world’s population, it is estimated that around 50 million individuals are living with dementia worldwide. The number of dementia cases might increase to 75 million by 2030 and almost triple by 2050 (1, 2). A marked gender difference exists in dementia. Women at the age of 65 years have more than a 55% greater risk of developing dementia than men (3, 4). Several preclinical and human studies hypothesized that decreased endogenous estrogen is associated with an increased risk of dementia, and the perimenopausal transition may be a window of opportunity to prevent cognitive impairment (5).

Possible neuroprotective and neurotrophic effects of estrogen at different life stages (6) inspired researchers to explore the relationship between cognition and reproductive factors, which include age at menarche, age at menopause, menopause type (natural or hysterectomy), reproductive period (i.e., the number of years between menarche and menopause), and pregnancy or parity number. Many epidemiologic studies have shown inconsistent findings due to differences in study design, age of participants, age at interview or follow-up, length of follow-up, and differences in adjustment for possible confounders. For example, younger age at menarche, shorter reproductive period, and more parities increased the risks of cognitive impairment, as well as dementia or Alzheimer’s disease (AD) (7–9), but other studies showed no or opposite association (10–12). Overall, most research has been conducted on older Western women who had longer reproductive periods than Chinese women (13). Little information is available for rural older women in China, although there were three studies in southern and eastern China in the past decade (14–16).

In this study, we conducted a population-based cross-sectional research to evaluate the relationship between reproductive factors and cognitive performance and also the associations with the risk of developing mild cognitive impairment (MCI) and dementia (including AD, dementia with Lewy bodies (DLB), and vascular dementia (VaD)) in women with natural menopause. Insofar as the reproductive period is a marker of long-term exposure to endogenous estrogen, we hypothesized that shorter exposure to endogenous estrogen (later age at menarche, earlier age at menopause, and shorter reproductive period) and more pregnancies/parities are associated with poor cognition, as well as elevated risks of MCI and dementia.

## Material and methods

### Participants

This cross-sectional study enrolled participants ≥65 years of age in 112 community primary healthcare centers selected from 949 villages in the rural Ji County in the northern part of Tianjin Province, northern China, between April 2019 and January 2020. This rural area has a mountainous terrain and most people are farmers and have the same lifestyle (17). The local medical practitioner in each village (who had worked there for over 5 years) was involved in identifying all individuals aged ≥65 years based on the date of birth provided on their residence certificate. A face-to-face questionnaire-based survey was conducted by senior MD students or medical staff in the local panel health centers, and a neurologist with special expertise in cognitive impairment re-reviewed the data in each region. All interviewers and experts received the same 1-week training on collecting information (consisting of demography, lifestyles, medical history, and reproductive factors), neuropsychological assessment, and diagnosis, and participated in a retraining course every 2 months.

**Figure 1:**
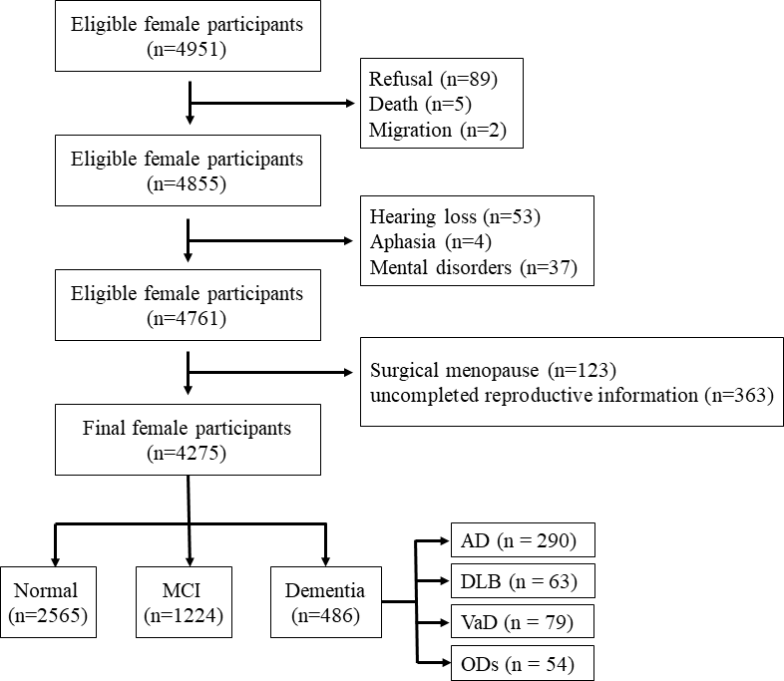
Flowchart of this study. MCI, mild cognitive impairment; AD, Alzheimer’s disease; DLB, dementia with Lewy bodies; VaD, vascular dementia; ODs, other dementias.

**Figure 1** shows the flow chart of study enrollment and exclusion. The total number of female participants aged ≥65 years in these communities was 4,951; however, due to refusal (*n* = 89), death (*n* = 5), migration (*n* = 2), hearing loss (*n* = 53), aphasia (*n* = 4), or mental disorders (including definite depression and anxiety, *n* = 37), a total of 4,761 completed the interview. As 123 participants with surgical menopause history and 363 participants with uncompleted reproductive information, 486 records were excluded, and 4,275 records were finally analyzed. The study was approved by the Committee for Medical Research Ethics at Tianjin Huanhu Hospital and the Tianjin Health Bureau (ID: 2019-40). Written informed consent was obtained from each subject either directly or from his/her guardian.

### Measures

In this study, all female participants had already reached menopause. Reproductive factors were collected in the reproductive history section, including age at menarche, age at menopause (natural or surgical), number of pregnancies, and number of parties. The reproductive period was calculated as age at menopause minus the age at menarche.

Information on other covariates was collected *via* the questionnaires, including age, education, marital status (single, married, divorced, or widowed), occupation (manual worker or nonmanual worker), living states (with spouse, with children, alone, or others), lifestyle (habits of smoking and drinking), and comorbidities (stroke, diabetes mellitus, heart disease, and hypertension) based on medical records or physical examination. In order to reduce recall bias, all the information above was collected with both participants and their caregivers present.

Cognitive assessment was conducted using the Mini-Mental State Examination (Chinese version) [(C-MMSE); range: 0–30 (no impairment)] (17), the Clinical Dementia Rating (CDR; 0, nondemented; 0.5, questionable dementia; 1, mild; 2, moderate; and 3, severe) (18), and the Activities of Daily Living (ADL, range 20 (no impairment) –80) scale (19). When C-MMSE scores were less than or equal to the cutoff point (≤17 for illiterate individuals, ≤20 for those with 1–6 years of education, and ≤24 for individuals with ≥7 years of education) or ADL was impaired (ADL > 20), we performed a second screening survey, with the detailed design described in a previous study (20).

The second phase to confirm subtypes of dementia included physical and neurological examinations, medical record review, and neuroimaging examinations (magnetic resonance imaging or computed tomography), ^11^C-PIB PET scan, and an ^18^F-FDG PET scan for those difficult to diagnose if possible. In this study, we diagnosed dementia based on the Diagnostic and Statistical Manual of Mental Disorders V criteria (DSM-5) (21). Probable AD was diagnosed according to the criteria of the National Institute on Aging and the Alzheimer Association workgroup (22), and VaD was diagnosed according to the National Institute of Neurologic Disorders and Stroke/Association International pour la Recherche et l’Enseignement en Neurosciences (NINDS-AIREN) criteria (23). A probable DLB diagnosis can be made with two or more core symptoms with or without indicative biomarkers, or only one core symptom with one or more indicative biomarkers using the criteria of McKeith et al. (24). The core clinical features of probable DLB were measured as below: visual hallucinations (VHs) were systematically assessed using a subscale of the Neuropsychiatric Inventory, with specific reference to the occurrence of VHs to exclude hallucinations in other modalities (e.g., auditory hallucinations) (25). Parkinsonism was considered present when the neurological examination showed extrapyramidal signs (tremor, bradykinesia, and/or rigidity) and was measured using UPDRS-III (26). The REM sleep behavior disorder (RBD) was rated as present according to caregiver’s reports stating the patient appeared to “act out” their dreams and was moving extensively during sleep and measured using the RBD screening questionnaire or polysomnography (27). Fluctuations including spontaneous alterations in cognition, attention, and arousal were rated positive according to the patient’s or caregiver’s complaints of changes during the day and over weeks, then assessed using the One Day Fluctuation Scale and the Clinical Assessment of Fluctuation scale, respectively (28). To distinguish DLB from Parkinson’s disease (PD) dementia, we excluded patients in whom cognitive impairment had occurred more than 1 year after they were diagnosed with the extrapyramidal syndrome. Based on the International Working Group on MCI diagnosis (29), we developed the following operational criteria for MCI: (i) cognitive decline was reported by the patient or caregiver or was found by an experienced clinician; (ii) objective evidence of a cognitive decline in one or more functional fields was confirmed on neuropsychological estimation; (iii) complex instrumental daily abilities could be slightly impaired, but basic ADL was relatively normal; and (iv) the patient’s symptoms did not match dementia.

### Data analysis

We examined the distribution of demographic characteristics, reproductive factors, and cognitive performance by reproductive period (≤30, 31–33, 34–36, and ≥37 years)/cognitive status ((healthy old adults, MCI, AD, DLB, VaD, and other dementias (ODs)) using descriptive statistics. A nonparametric test was used for continuous variables consistent with nonnormal applications (age, education, reproductive factors, and C-MMSE and ADL scores), and *χ* ^2^ tests for categorical variables (marital state, occupation, living status, lifestyles, and comorbidities). The prevalence rates of MCI, dementia, AD, DLB, and VaD were calculated by prevalence (%) with 95% confidence intervals (CIs).

Correlation and logistic regressions were used to estimate the associations between reproductive factors and cognition. Relationships between reproductive factors and scores of C-MMSE were determined by Spearman’s correlation with a crude model, and partial correlations with adjusting age and education in all participants and women with MCI, dementia, AD, DLB, or VaD. We performed logistic regression analyses with reproductive factors as the predictor and MCI/dementia/AD/DLB/VaD as the outcome compared with healthy old adults. Logistic regression models were used to estimate the odds ratio (OR) for each outcome associated with self-reported reproductive factors, adjusting for potential confounders. All data were analyzed using IBM SPSS Statistics for Windows (Version 25.0; IBM Corp., Armonk, NY, USA), with *p* < 0.05 considered significant.

## Results

### General characteristics of participants

The general characteristics of 4,275 natural postmenopausal women are shown in **Tables 1**, **2**. The mean (± standard deviation) age was 74.32 (± 5.72) years, with an average of 32.97 (± 5.03) years of reproductive duration.

**Table 1:** General characteristics of participants.

Characteristics	All samples (*n* = 4,275)	Reproductive duration (years)				*p*-values
		≤30 (*n* = 1,143)	31–33 (*n* = 1,176)	34–36 (*n* = 1,042)	≥37 (*n* = 914)	
**Age (years, mean** ± **SD)**	74.32 ± 5.72	74.41 ± 5.57	74.92 ± 5.90	74.11 ± 5.66	73.68 ± 5.65	d, e
**Education (years, mean** ± **SD)**	4.90 ± 4.41	4.01 ± 4.03	4.71 ± 4.36	5.28 ± 4.32	5.83 ± 4.77	a, b, c, d, e
**Marital status (*n*, %)**						
Married	3,078 (72.0%)	801 (70.1%)	812 (69.0%)	773 (74.2%)	692 (75.7%)	None
Single	7 (0.2%)	2 (0.2%)	2 (0.2%)	1 (0.1%)	2 (0.2%)	
Divorced	48 (1.1%)	13 (1.1%)	10 (0.9%)	14 (1.3%)	11 (1.2%)	
Widowed	1,142 (26.7%)	327 (28.6%)	352 (29.9%)	254 (24.4%)	209 (22.9%)	
**Occupation (*n*, %)**						
Manual worker	2,471 (57.8%)	768 (67.2%)	703 (59.8%)	552 (53.0%)	448 (49.0%)	a, b, c, d, e
Nonmanual worker	1,804 (42.2%)	375 (32.8%)	473 (40.2%)	490 (47.0%)	466 (51.0%)	
**Living states (*n*, %)**						
With children	998 (23.3%)	282 (24.7%)	285 (24.2%)	245 (23.5%)	186 (20.4%)	None
With spouse	2,714 (63.5%)	722 (63.2%)	726 (61.7%)	677 (65.0%)	589 (64.4%)	
Alone	521 (12.2%)	132 (11.5%)	155 (13.2%)	105 (10.1%)	129 (14.1%)	
Others^a^	42 (1.0%)	7 (0.6%)	10 (0.9%)	15 (1.4%)	10 (1.1%)	
**Lifestyles (*n*, %)**						
Smoking (yes)	192 (4.5%)	56 (4.9%)	68 (5.8%)	36 (3.6%)	31 (3.4%)	None
Drinking (yes)	149 (3.5%)	45 (3.9%)	48 (4.1%)	25 (2.4%)	31 (3.4%)	None
**Comorbidities (yes, *n*, %)**						
Stroke	434 (10.2%)	115 (10.1%)	114 (9.7%)	100 (9.6%)	105 (11.5%)	None
DM	684 (16.0%)	151 (13.2%)	179 (15.2%)	198 (19.0%)	156 (17.1%)	b
Heart disease	755 (17.7%)	218 (19.2%)	207 (17.6%)	181 (17.4%)	149 (16.3%)	None
Hypertension	2,285 (53. 5%)	632 (55.3%)	621 (52.8%)	554 (53.2%)	478 (52.3%)	None
**Reproductive factors (mean** ± **SD)**						
Age at menarche (years)	16.36 ± 2.25	17.58 ± 2.31	16.81 ± 1.86	15.87 ± 1.82	14.94 ± 2.02	a, b, c, d, e, f
Age at menopause (years)	49.33 ± 4.52	44.50 ± 3.87	48.90 ± 0.79	50.81 ± 1.88	54.11 ± 2.92	a, b, c, d, e, f
Reproductive period (years)	32.97 ± 5.03	26.93 ± 3.49	32.30 ± 0.79	34.94 ± 0.81	39.18 ± 2.25	a, b, c, d, e, f
Num. of pregnancies	3.19 ± 1.45	3.25 ± 1.40	3.27 ± 1.44	3.10 ± 1.40	3.10 ± 1.41	None
Num. of parities	2.60 ± 1.20	2.73 ± 1.15	2.74 ± 1.28	2.50 ± 1.15	2.40 ± 1.18	b, c, d, e
**C-MMSE (mean** ± **SD)**	24.53 ± 5.23	23.96 ± 5.19	23.78 ± 6.03	25.29 ± 4.71	25.36 ± 4.48	b, c, d, e
**ADL (mean** ± **SD)**	21.67 ± 5.46	21.79 ± 5.22	22.46 ± 7.11	21.12 ± 4.06	21.12 ± 4.47	b, c, d, e

SD, standard deviation; DM, diabetes mellitus; Num., number; C-MMSE, Chinese Mini-Mental State Examination; ADL, the activities of daily life.

a Others mean the participants lived with other relatives or nannies or lived in a nursing home.

The p-values with significant difference (p < 0.05/6 = 0.008 after Bonferroni correction) were recorded as follows: (a) group: (≤30 years of reproductive duration) vs. group (31–33 years of reproductive duration); (b) group (≤30 years of reproductive duration) vs. group (34–36 years of reproductive duration); (c) group (≤30 years of reproductive duration) vs. group (≥37 years of reproductive duration); (d) group (31–33 years of reproductive duration) vs. group (34–36 years of reproductive duration); (e) group (31–33 years of reproductive duration) vs. group (≥37 years of reproductive duration); and (f) group (34–36 years of reproductive duration) vs. group (≥37 years of reproductive duration). Those variables without significant difference were not recorded.

**Table 2:** General characteristics of participants by diagnosis.

Characteristics	Healthy old adults (*n* = 2,565)	MCI (*n* = 1,224)	AD (*n* = 290)	DLB (*n* = 63)	VaD (*n* = 79)	ODs (*n* = 54)
**Age (years, mean** ± **SD)**	73.63 ± 5.44	74.57 ± 5.69^***^	77.12 ± 6.28^***^	75.90 ± 6.10^**^	79.30 ± 5.84^***^	77.11 ± 5.36^***^
**Education (years, mean** ± **SD)**	5.27 ± 4.35	4.68 ± 4.48^***^	3.74 ± 4.50^***^	3.17 ± 3.71^***^	2.80 ± 3.32^***^	3.68 ± 4.54^**^
**Marital status (*n*, %)**						
Married	1,926 (75.1%)	848 (69.3%)^***^	181 (62.4%)^***^	45 (71.4%)	46 (58.2%)^**^	32 (59.3%)^**^
Single	5 (0.2%)	1 (0.1%)	1 (0.3%)	0 (0.0%)	0 (0.0%)	0 (0.0%)
Divorced	20 (1.2%)	16 (1.3%)	2 (0.7%)	0 (0.0%)	0 (0.0%)	0 (0.0%)
Widowed	604 (23.5%)	359 (29.3%)^***^	106 (36.6%)^***^	18 (28.6%)	33 (41.8%)^**^	22 (40.7%)^**^
**Occupation (*n*, %)**						
Manual worker	1,498 (58.4%)	671 (54.8%)	176 (60.7%)	552 (53.0%)	47 (59.5%)	36 (66.7%)
Nonmanual worker	1,067 (41.6%)	553 (45.2%)	114 (39.3%)	20 (31.7%)	32 (40.5%)	18 (33.3%)
**Living states (*n*, %)**						
With children	533 (20.8%)	298 (24.3%)	98 (33.8%)	19 (30.2%)	29 (36.7%)	21 (38.9%)
With spouse	1,722 (67.1%)	738 (60.3%)	150 (51.7%)	36 (57.1%)	43 (54.4%)	25 (46.3%)
Alone	289 (11.3%)	173 (14.1%)	39 (13.4%)	8 (12.7%)	5 (6.3%)	7 (13.0%)
Others^a^	21 (0.8%)	15 (1.2%)	3 (1.0%)	0 (0.0%)	2 (2.5%)	1 (1.9%)
**Lifestyles (*n*, %)**						
Smoking (yes)	110 (4.3%)	60 (4.9%)	11 (3.8%)	5 (7.9%)	3 (3.8%)	3 (5.6%)
Drinking (yes)	91 (3.5%)	46 (3.8%)	9 (3.1%)	2 (3.2%)	1 (1.3%)	0 (0.0%)
**Comorbidities (yes, *n*, %)**						
Stroke	229 (8.9%)	120 (9.8%)	31 (10.7%)	9 (14.3%)	31 (39.2%)^***^	14 (25.9%)^***^
DM	392 (15.3%)	210 (17.2%)	30 (10.3%)	19 (30.2%)^**^	31 (39.2%)^***^	2 (3.7%)^***^
Heart disease	446 (17.4%)	209 (17.1%)	27 (9.3%)	15 (23.8%)	37 (46.8%)^***^	21 (38.9%)^***^
Hypertension	1,369 (53. 4%)	651 (53.2%)	120 (41.4%)	37 (58.7%)	73 (92.4%)^***^	35 (64.8%)
**Reproductive factors (mean** ± **SD)**						
Age at menarche (years)	16.35 ± 2.22	16.26 ± 2.28	16.64 ± 2.34	16.98 ± 2.09^*^	16.36 ± 2.46	16.64 ± 2.64
Age at menopause (years)	49.59 ± 4.38	32.76 ± 5.00^***^	48.35 ± 4.47^***^	49.35 ± 5.42	48.11 ± 4.51^**^	50.02 ± 5.05
Reproductive period (years)	33.23 ± 4.81	26.93 ± 3.49^**^	31.95 ± 4.68^***^	32.48 ± 5.09	31.99 ± 4.68^**^	33.32 ± 4.90
Num. of pregnancies	3.08 ± 1.35	3.27 ± 1.44^***^	3.41 ± 1.57^**^	3.64 ± 1.76^**^	3.85 ± 1.77^***^	3.51 ± 1.46^**^
Num. of parities	2.48 ± 1.11	2.68 ± 1.22^***^	2.98 ± 1.39^***^	3.10 ± 1.60^***^	3.44 ± 1.53^***^	3.04 ± 1.36^***^
**C-MMSE (mean** ± **SD)**	27.37 ± 5.23	22.76 ± 2.86^***^	14.00 ± 5.15^***^	14.83 ± 5.67^***^	13.39 ± 5.54^***^	14.24 ± 5.12^***^
**ADL (mean** ± **SD)**	20.00 ± 0.00	20.00 ± 0.00	34.18 ± 7.46^***^	33.73 ± 8.45^***^	36.27 ± 9.96^***^	36.02 ± 10.53^***^

The comparison between healthy old adults, MCI, AD, DLB, VaD, and ODs were calculated respectively, and the significant p-values were recorded as ^*^p < 0.05; ^**^p < 0.01; ^***^p < 0.001.

a Others mean the participants lived with other relatives or nannies or lived in a nursing home.

SD, standard deviation; MCI, mild cognitive impairment; AD, Alzheimer’s disease; DLB, dementia with Lewy bodies; VaD, vascular dementia; ODs, other dementias; DM, diabetes mellitus; Num., number; C-MMSE, Chinese Mini-Mental State Examination; ADL, the activities of daily life.

**Figure 2:**
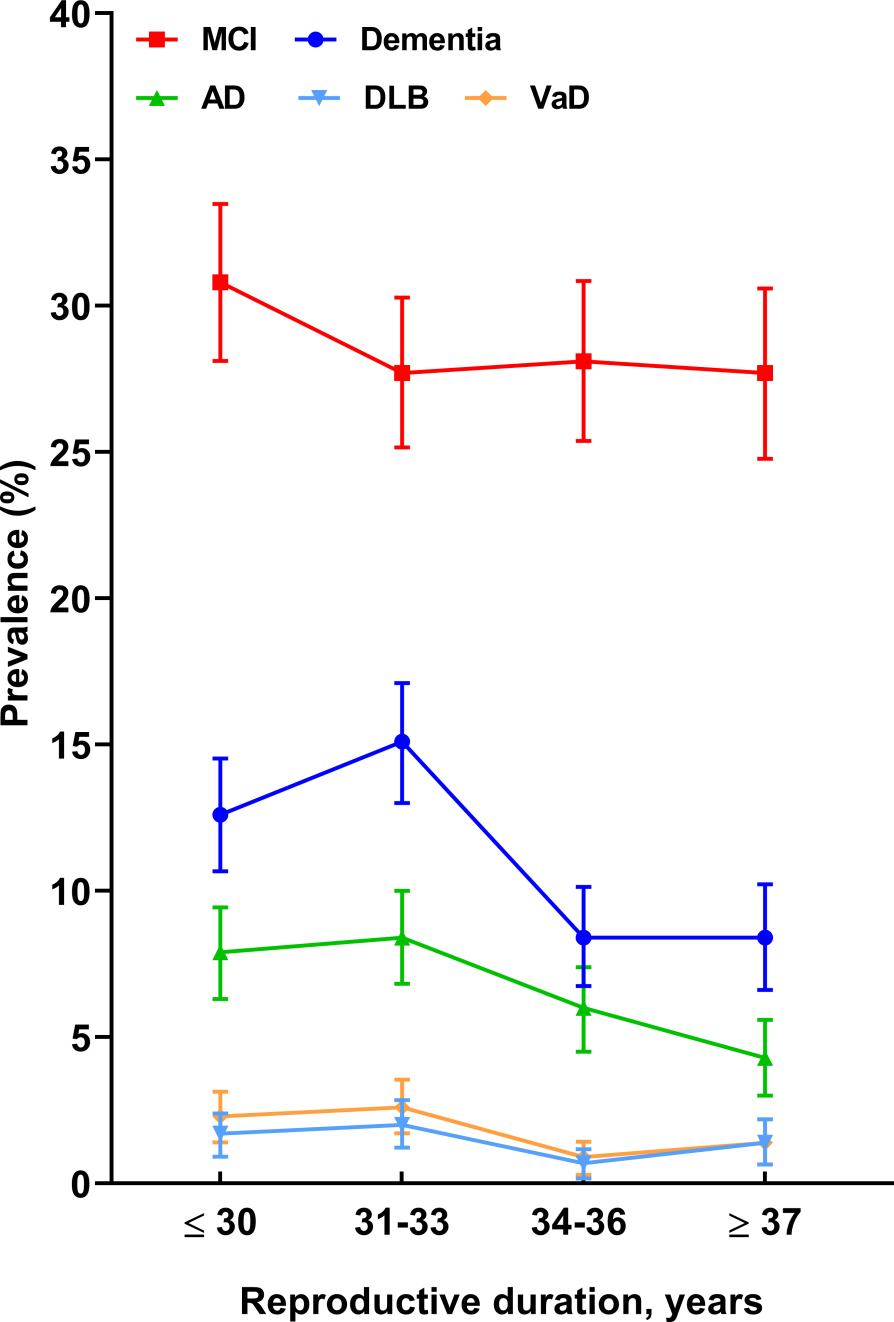
The prevalence rates (with 95% CIs) of cognitive impairment across the reproductive period. 95% CIs, 95% confidential intervals; MCI, mild cognitive impairment; AD, Alzheimer’s disease; DLB, dementia with Lewy bodies; VaD, vascular dementia.

Overall, 28.6% (95% CI: 27.3–30.0%) and 11.4% (95% CI: 10.4–12.3%) of women were diagnosed with MCI and dementia, respectively. Women with ≤30 years of reproductive duration had the highest prevalence rate of MCI (30.8%, 95% CI: 28.1–33.5%), and those with 31–33 years had the highest prevalence rate of dementia (15.1%, 95% CI: 13.0–17.1%), as well as AD (8.4%, 95% CI: 6.8–10.0%), DLB (2.0%, 95% CI: 1.2–2.9%), and VaD (2.6%, 95% CI: 1.7 –3.6%) (**Figure 2**).

### Association between reproductive characteristics and cognitive performance

**Table 3** shows the correlation coefficients between reproductive factors and C-MMSE scores. The age at menopause (adjusted *r* = 0.070, *p* < 0.001), reproductive period (adjusted *r* = 0.053, *p* = 0.001), number of pregnancies (adjusted *r* = −0.042, *p* = 0.007), and number of parities (adjusted *r* = −0.068, *p* < 0.001) were correlated with C-MMSE scores in Model 2. There were similar findings in women with MCI (age at menopause, crude *r* = −0.244, *p* < 0.001; reproductive period, crude *r* = 0.150, *p* < 0.001; number of pregnancies, crude *r* = −0.123, *p* < 0.001; and number of parities, crude *r* = −0.251, *p* < 0.001). Additionally, a significant negative correlation (adjusted *r* = −0.486, *p* < 0.001) was found between age at menopause and C-MMSE scores in women with DLB.

**Table 3:** Correlation coefficients of reproductive factors and MMSE score (Spearman’s rho).

Reproductive factors	C-MMSE scores					
	All samples		MCI		Dementia	
	Model 1	Model 2	Model 1	Model 2	Model 1	Model 2
**Age at menarche**	−0.092^***^	0.023	−0.244^***^	0.048	−0.178^***^	0.017
**Age at menopause**	0.103^***^	0.070^***^	0.037	0.024	0.033	0.011
**Reproductive period**	0.129^***^	0.053^**^	0.150^***^	0.001	0.054	0.017
**Number of pregnancies**	−0.124^***^	−0.042^**^	−0.123^***^	−0.009	−0.401	0.002
**Number of parities**	−0.217^***^	−0.068^***^	−0.251^***^	0.002	−0.121^*^	−0.035
	**AD**		**DLB**		**VaD**	
	**Model 1**	**Model 2**	**Model 1**	**Model 2**	**Model 1**	**Model 2**
**Age at menarche**	−0.119	0.048	−0.627***	−0.486***	−0.109	−0.005
**Age at menopause**	0.038	0.034	−0.038	0.035	0.057	−0.076
**Reproductive period**	0.037	0.009	0.115	0.196	0.024	−0.064
**Number of pregnancies**	−0.027	−0.009	−0.159	−0.227	−0.046	−0.084
**Number of parities**	−0.061	−0.037	−0.224	−0.195	−0.267*	−0.192

Spearman’s correlation analysis was used to calculate the correlation between reproductive factors and C-MMSE scores in Model 1, and Model 2 was performed by adjusting age and education with partial correlation. ^*^p < 0.05; ^**^p < 0.01; ^***^p < 0.001.

C-MMSE, Mini-Mental State Examination (Chinese version); MCI, mild cognitive impairment; AD, Alzheimer’s disease; DLB, dementia with Lewy bodies; VaD, vascular dementia.

The logistic regressions showed the associations between reproductive factors and risks of MCI or dementia, especially AD, DLB, and VaD (**Table 4**). Greater age at menopause (OR = 0.973, 95% CI: 0.058–0.989, *p* = 0.001 in MCI; OR = 0.964, 95% CI: 0.941–0.988, *p* = 0.003 in dementia; and OR = 0.949, 95% CI: 0.921–0.978, *p* = 0.001 in AD) and long reproductive period (OR = 0.982, 95% CI: 0.968–0.996, *p* = 0.011 in MCI; OR = 0.969, 95% CI: 0.949–0.990, *p* = 0.004 in dementia; and OR = 0.960, 95% CI: 0.935–0.085, *p* = 0.002 in AD) significantly decreased the risk of MCI, dementia, and AD after adjusting for all demographic and clinical variables. Meanwhile, a larger number of parities significantly increased the risks of MCI (OR = 1.111, 95% CI: 1.039–1.187, *p* = 0.002), dementia (OR = 1.162, 95% CI: 1.061–1.271, *p* = 0.001), AD (OR = 1.131, 95% CI: 1.010–1.266, *p* = 032), DLB (OR = 1.238, 95% CI: 1.003–1.528, *p* = 0.047), and VaD (OR = 1.288, 95% CI: 1.080–1.536, *p* = 0.005).

**Table 4:** Association between reproductive factors and risks of cognitive impairment by logistic regressions (ORs with 95% CI).

	Age at menarche	Age at menopause	Reproductive period	Num. of pregnancies	Num. of parities
**MCI**					
**Crude**	0.982 (0.952–1.013)	0.972 (0.957–0.987)^***^	0.981 (0.967–0.994)^**^	1.105 (1.051–1.161)^***^	1.163 (1.096–1.234)^***^
**Model 1**	0.965 (0.934–0.997)^*^	0.974 (0.959–0.989)^**^	0.986 (0.972–0.999)^*^	1.069 (1.015–1.126)^**^	1.101 (1.031–1.175)^**^
**Model 2**	0.982 (0.950–1.016)	0.973 (0.058–0.989)^**^	0.982 (0.968–0.996)^*^	1.067 (1.012–1.124)^*^	1.111 (1.039–1.187)^**^
**Dementia**					
**Crude**	1.058 (1.009–1.109)^*^	0.954 (0.932–0.976)^***^	0.957 (0.938–0.976)^***^	1.235 (1.155–1.321)^***^	1.466 (1.355–1.585)^***^
**Model 1**	1.013 (0.964–1.065)	0.962 (0.940–0.985)^**^	0.971 (0.951–0.991)^**^	1.074 (0.998–1.155)	1.180 (1.080–1.289)^***^
** Model 2**	1.583 (0.983–1.090)	0.964 (0.941–0.988)^**^	0.969 (0.949–0.990)^**^	1.061 (0.985–1.142)	1.162 (1.061–1.271)^**^
**AD**					
**Crude**	1.058 (0.997–1.123)	0.941 (0.914–0.968)^***^	0.949 (0.926–0.972)^***^	1.177 (1.080–1.282)^***^	1.391 (1.262–1.533)^***^
**Model 1**	1.020 (0.958–1.086)	0.949 (0.922–0.977)^***^	0.961 (0.937–0.986)^**^	1.029 (0.939–1.129)	1.133 (1.014–1.265)^*^
**Model 2**	1.036 (0.972–1.105)	0.949 (0.921–0.978)^***^	0.960 (0.935–0.085)^**^	1.028 (0.936–1.129)	1.131 (1.010–1.266)^*^
**DLB**					
**Crude**	1.132 (1.004–1.277)^*^	0.987 (0.929–1.050)	0.969 (0.922–1.019)	1.296 (1.104–1.522)^**^	1.474 (1.230–1.766)^***^
**Model 1**	1.066 (0.940–1.209)	0.994 (0.936–1.056)	0.986 (0.938–1.037)	1.186 (0.999–1.408)	1.268 (0.817–0.951)^*^
**Model 2**	1.009 (0.965–1.251)	0.994 (0.934–1.057)	0.980 (0.932–1.032)	1.160 (0.971–1.386)	1.238 (1.003–1.528)^*^
**VaD**					
**Crude**	1.001 (0.895–1.119)	0.932 (0.885–0.981)^**^	0.951 (0.910–0.993)^*^	1.400 (1.219–1.608)^***^	1.730 (1.482–2.020)^***^
**Model 1**	0.945 (0.839–1.064)	0.947 (0.898–0.999)^*^	0.973 (0.929–1.019)	1.059 (0.995–1.343)	1.288 (1.080–1.536)^**^
**Model 2**	0.991 (0.867–1.132)	0.940 (0.887–0.996)^*^	0.963 (0.916–1.013)	1.118 (0.949–1.318)	1.224 (0.994–1.508)

Logistic regressions were performed to measure the risks of MCI, dementia, AD, DLB, and VaD compared with healthy old adults in five subgroups. Model 1 was adjusted by age and education; Model 2 was adjusted by age, education, marital status, occupation, living states, lifestyles, and all comorbidities mentioned in **Table 1**. ^*^p < 0.05; ^**^p < 0.01; ^***^p < 0.001.

ORs, odds ratios; CI, confidence interval; MCI, mild cognitive impairment; AD, Alzheimer’s disease; DLB, dementia with Lewy bodies; VaD, vascular dementia.

## Discussion

Consistent with our original hypothesis, this population-based study indicated that younger age at menopause, shorter reproductive period, and more pregnancies or parities were associated with a higher risk of MCI, dementia, as well as AD and DLB in women with natural menopause, after adjustment for multiple confounding variables. Overall, these findings support the hypothesis that longer endogenous estrogen exposure reduces the risk of cognitive impairment.

### Prior literature

Our findings are consistent with two large epidemiological studies of reproductive factors and cognition in mainland China (14, 15). The first cross-sectional study, which included 11,094 naturally postmenopausal multiparous older Chinese (≥50 years) women from the Guangzhou Biobank Cohort Study, found that longer reproductive period and lower parity were associated with better cognition (14). Another study including 4,796 postmenopausal women, based on data from the Zhejiang Major Public Health Surveillance Program, also found that a longer reproductive period and lower pregnancy rate were associated with decreased risk of CI (15). There were similar findings in several studies among older Asian and Western women (9, 12, 30–32). In Gilsanz’s prospective cohort with 15,754 female members, those who had menarche at ≥ 16 years, natural menopause at age <47.4 years, and reproductive period of <34.4 years were associated with an elevated risk of dementia (8). Taiwan Biobank data also indicated that late menarche was associated with poor cognitive function (16). A meta-analysis showed that older age at menopause and a longer reproductive period were associated with a lower risk of dementia (33, 34).

However, findings in other studies have been inconsistent. There was no evidence of an association between the reproductive period and dementia in the study of Prince etal. (11) or in a Chinese study of 520 postmenopausal women with continuous data (16). In a population-based sample of women followed up over 44 years, a longer reproductive period and later menopause were related to an increased risk of dementia and AD, but this was not associated with age at menarche and the number of pregnancies among women with natural menopause (35). However, the Rotterdam study found that longer reproductive periods were associated with increased dementia risk among women with at least one *APOE* ϵ4 allele, but there was no association among noncarriers (10). Several differences between these studies might explain the differing results. Studies, where a longer reproductive period was not associated with increased dementia risk, had a higher mean reproductive period (35.9 years in the Rotterdam cohort (10), and 36.0 years in Taiwan, China (16)) or were conducted in white-race, high-income countries (10, 35). Additionally, the study design (cross-sectional or prospective) and the factors in adjusted models in the analyses differed. Old age and low educational level are traditional risks for cognitive impairment (2), and the lower education and older age in this study might explain the inconsistency.

Estrogen can modulate nigrostriatal dopaminergic activity. We found that later age at menarche, more pregnancies (in crude models), and more parities (in Model 2) increased the risks of DLB. To date, no studies have explored the relationship between estrogen status and the risk of DLB. Several studies were conducted in PD, showing the same equilibrium of alpha-synuclein conformations with DLB, but the findings are conflicting (36–38). The mechanism relating to reproductive factors and PD is not clear. Even though there is a genetic component to the reproductive factors, external influences likely influence both the reproductive factors and PD risk. More large-sample cohort research is needed. In addition, associations between natural vs. surgical menopause and risks of cognitive impairment are controversial. Surgical menopause is likely to result in an earlier age at menopause and a shorter reproductive period (39, 40), which may be associated with a faster decline in global cognition, specifically episodic memory, semantic memory, verbal fluency, executive function, and accumulation of AD neuropathology (39–41). Although observational evidence suggests that the natural menopausal transition is not associated with substantial changes in cognitive abilities, estrogen-containing hormone therapy has little effect on cognition during midlife or postmenopause (42).

### Possible mechanisms

Our epidemiologic findings support the hypothesis of the neuroprotective effects of estrogen. Postmenopause is characterized by notably reduced estrogen and elevated follicle-stimulating hormone (FSH) levels. Decreased estradiol or progesterone after menopause weakens the effects of neuronal resilience and repair and promotes inflammation, apoptosis, and tau hyperphosphorylation (43). The FSH acts directly on hippocampal and cortical neurons to accelerate amyloid-β and Tau deposition and impair cognition in mice displaying features of AD. Current research shows that blocking FSH action in these mice abrogates the AD-like phenotype by inhibiting the neuronal C/EBPβ–δ-secretase pathway (44). More parity brought longer periods of anxiety, depression, and other emotional disorders, as well as sleep deprivation due to feeding and nighttime care, which may be associated with an increased risk of cognitive impairment (45, 46).

### Strengths and limitations

The main strengths of our study were the large population in rural northern China and the detailed collection of information on participants (demographic, dietary, lifestyle factors, and medical history) to investigate the associations between reproductive factors with cognitive impairment, particularly AD and DLB. Nevertheless, several limitations should be noted. First, recall bias remains regarding the retrospective exposure variables, although we investigated both participants and their caregivers. In addition, the sample comprises Chinese women living in northern China, limiting the possibility of generalizing to other populations. Another limitation is the lack of objective biomarkers, such as endogenous estrogen and AD biomarkers. Finally, we cannot determine any causal relationship in this cross-sectional study, and additional large prospective studies including diverse populations are needed to reflect the increasing diversity of the aging population in China.

## Conclusions

We found that natural menopause females with a younger age at menopause, a shorter reproductive period, and more pregnancies or parities had a higher risk of MCI, dementia, AD, and DLB. Women bear a large and disproportionate burden of dementia, so further research is required on the reproductive factors to explore the relative optimal reproductive period and the number of pregnancy/parities, so as to provide more protection for women’s health and provide a reference for the formulation of national fertility policies.

## Data availability statement

The raw data supporting the conclusions of this article will be made available by the authors, without undue reservation.

## Ethics statement

The study was approved by the Committee for Medical Research Ethics at Tianjin Huanhu Hospital and the Tianjin Health Bureau (ID: 2019-40). The patients/participants provided their written informed consent to participate in this study.

## Author contributions

Conceptualization: YJ. Methodology: YJ. Software: FW. Validation: Z-HS and YJ. Formal analysis: FW and Z-CC. Investigation: all authors. Re-sources: Z-HS, X-DW, and YJ. Data curation: SL. Writing—original draft preparation: H-TX, and J-HG. Writing—review and editing: H-TX, J-HG, and Z-HS. Visualization: X-DW. Supervision: SL. Project administration: YJ. Funding acquisition: YJ. All authors have read and agreed to the published version of the manuscript.

## Funding

This work was supported by the National Natural Science Foundation of China (grant number 82171182) and Science and Technology Project of Tianjin Municipal Health Committee (grant number ZC20121 and KJ20048).

## Acknowledgments

The authors are grateful to all those who participated in this study and wish to acknowledge the valuable assistance obtained from all specialized physicians. We sincerely gratitude Jing Li (Tianjin Huanhu Hospital, Tianjin, China), Wenzheng Hu (Beijing Tiantan Hospital, Capital Medical University, Beijing, China), Xiyu Li (Tianjin medical university, Tianjin, China), Han Zhu (Tianjin medical university, Tianjin, China), Xiaoshan Du (Tianjin medical university, Tianjin, China), Wenjing Zhou (Tianjin medical university, Tianjin, China), and Lingyun Ma (Beijing Tiantan Hospital, Capital Medical University, Beijing, China) for the data collection and input.

## Conflict of interest

The authors declare that the research was conducted in the absence of any commercial or financial relationships that could be construed as a potential conflict of interest.

## Publisher’s note

All claims expressed in this article are solely those of the authors and do not necessarily represent those of their affiliated organizations, or those of the publisher, the editors and the reviewers. Any product that may be evaluated in this article, or claim that may be made by its manufacturer, is not guaranteed or endorsed by the publisher.
